# Current Uncertainties and Future Challenges Regarding NAD+ Boosting Strategies

**DOI:** 10.3390/antiox11091637

**Published:** 2022-08-24

**Authors:** Borut Poljšak, Vito Kovač, Irina Milisav

**Affiliations:** 1 Laboratory of Oxidative Stress Research, Faculty of Health Sciences, University of Ljubljana, Zdravstvena pot 5, SI-1000 Ljubljana, Slovenia; 2 Faculty of Medicine, Institute of Pathophysiology, University of Ljubljana, Zaloska 4, SI-1000 Ljubljana, Slovenia

**Keywords:** nicotinamide adenine dinucleotide (NAD+), NAD+ boosters, NAD+ precursors, safety, side effects

## Abstract

Precursors of nicotinamide adenine dinucleotide (NAD+), modulators of enzymes of the NAD+ biosynthesis pathways and inhibitors of NAD+ consuming enzymes, are the main boosters of NAD+. Increasing public awareness and interest in anti-ageing strategies and health-promoting lifestyles have grown the interest in the use of NAD+ boosters as dietary supplements, both in scientific circles and among the general population. Here, we discuss the current trends in NAD+ precursor usage as well as the uncertainties in dosage, timing, safety, and side effects. There are many unknowns regarding pharmacokinetics and pharmacodynamics, particularly bioavailability, metabolism, and tissue specificity of NAD+ boosters. Given the lack of long-term safety studies, there is a need for more clinical trials to determine the proper dose of NAD+ boosters and treatment duration for aging prevention and as disease therapy. Further research will also need to address the long-term consequences of increased NAD+ and the best approaches and combinations to increase NAD+ levels. The answers to the above questions will contribute to the more efficient and safer use of NAD+ boosters.

## 1. Introduction

The nicotinamide adenine dinucleotide molecule (NAD+) is a redox coenzyme in electron transfer during oxidation–reduction reactions in mitochondria to generate ATP [1] and as a cofactor for NAD+-dependent enzymes, such as sirtuins (SIRT1-7 in mammals), poly (ADP-ribose) polymerases (PARPs), COOH-terminal binding protein (CtBP), cyclic ADP-ribose (ADPR) synthetases, glycoproteins CD38 and CD157, and many other NAD+-dependent enzymes [2,3]. NAD+ is required for over 500 enzymatic reactions and is involved in numerous evolutionarily conserved signaling pathways that include DNA repair, apoptosis, genomic signaling, endocrine signaling, senescence, proliferation, inflammation, mitochondrial function, lipid and glucose homeostasis [3].

NAD+ levels decrease during aging because of decreased synthesis and increased consumption of NAD+ due to (i) increased DNA damage and damage repair (activation of PARPs and sirtuins), (ii) increased secretion of inflammatory factors and inflammatory processes that activate CD38 or cause a defect in the enzyme nicotinamide phosphoribosyltransferase (NAMPT)-mediated NAD+ biosynthesis [4,5], and (iii) alcohol metabolism, resulting in an overall tissue-specific depletion in the concentration of NAD+ [6,7,8,9,10,11,12,13,14]. NAD+ is also used during NAD+ kinase phosphorylation where NADP+ is generated, which in turn is reduced by dehydrogenases to generate NADPH [15]. In summary, NAD+ levels decrease with age due to increased DNA damage, oxidative stress, and chronic inflammation, which dysregulate NAD metabolism by activating CD38 and PARPs or by inhibiting NAMPT [10,16]. As a result of decreased NAD+ levels, a reduction in cellular energy production and DNA repair, as well as altered genomic signaling, leads to aging and an increased incidence of chronic diseases [2,7,8,9]. Indeed, during aging and in many age-associated diseases, a decreased NAD+ availability was observed [6,7,8,9]. On the other hand, by increasing the availability of NAD+ and by preventing NAD+ degradation, aging and age-associated diseases could be modulated, delayed, and perhaps even reversed [6,7,8,9]. Scientific studies reports imply that besides life extension [17,18,19,20] and phenotypes consistent with an aging delay [21], rising NAD+ levels influence a number of different diseases and conditions, including metabolic syndrome, type 2 diabetes, and/or insulin sensitivity [5,17,22,23], cancer [24,25,26,27,28], cardiovascular disease [29,30,31,32,33,34], neurodegeneration [35,36,37], renal function [38,39], Alzheimer’s disease [40,41], and reduce inflammation [18,42,43], as well as help to prevent obesity [5,22,44,45,46]. NAD+ depletion was reported in neurological diseases as an increase in the ratio of NMN to NAD+ triggers axonal degeneration due to Sterile alpha and Toll/interleukin-1 receptor motif-containing 1 (SARM1) hydrolyzilation of NAD+ [47]. Namely, SARM1 is a neuronally expressed NAD+ glycohydrolase whose activity is increased in response to stress resulting in degeneration and programmed axon death (Wallerian degeneration) [48,49,50]. NAD+ deficiency is also associated with retinal degeneration and human vision impairment as deficits in NAD+ bioavailability promote senescence in retinal cells [51,52].

## 2. Increasing NAD+ Levels

**Figure 1:**
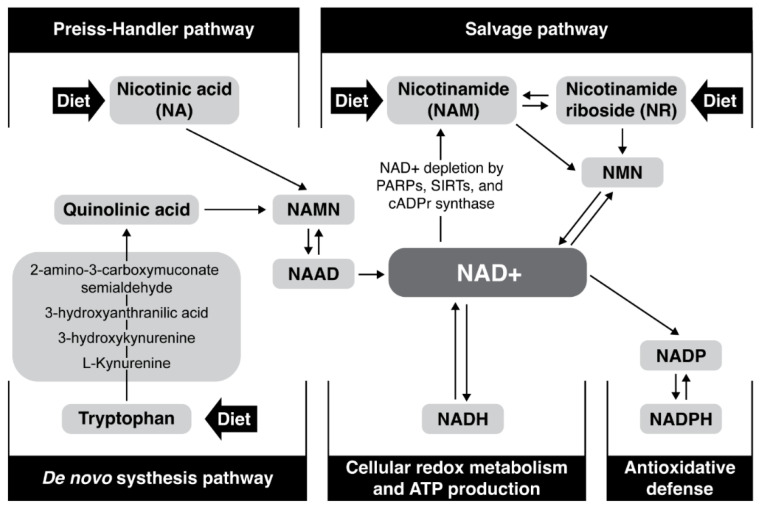
Major NAD+ biosynthesis, consumption, and redox-cycling routes.

Tryptophan, nicotinic acid (pyridine-3 carboxylic acid), nicotinamide (nicotinic acid amide), nicotinamide mononucleotide (NMN) and nicotinamide riboside (NR) are precursors of NAD+ in mammals. NAD+ can be produced and reused by three pathways in humans (Figure 1): (1) de novo synthesis (from L-tryptophan), (2) Preiss–Handler pathway (from nicotinic acid or nicotinic acid ribose), and (3) salvage pathway (from nicotinamide mononucleotide, niacinamide/nicotinamide (NAM) and nicotinamide riboside) [53,54,55,56]. De novo synthesis from tryptophan is an eight-step pathway; there are three steps from NA, while two steps are required for its synthesis from Nam or NR [57,58]. Different cell types preferentially use different NAD+ biosynthesis pathways under non-stress conditions [59,60]. For example, liver cells depend largely on de novo biosynthesis, while kidney cells use a combination of the Preiss–Handler and de novo pathways, and the salvage pathway is generally used to recycle NAM generated by NAD+-consuming reactions. Pharmacologic restoration of NAD+ is at the moment intensively investigated and approaches include NAD+-replacement therapy with NAD+ precursors such as NR, NMN, and NA supplementation [4,22] or by halting NAD+ utilization by poly-ADP-ribose polymerase (PARP) and CD 38 inhibitors [61,62,63,64,65,66]. Recently, a reduced form of nicotinamide riboside NRH (1-[(2R,3R,4S,5R)-3,4-dihydroxy-5-(hydroxymethyl)tetrahydrofuran-2-yl]-4H-pyridine-3-carboxamide) was discovered to function as an orally bioavailable NAD+ precursor with the potential to significantly increase NAD+ levels and to overcome the degradability of NR [67].

## 3. NAD+ Precursors and Boosters

NAD+ boosters include NAD+ precursors, pharmacological inhibitors of enzymes consuming NAD+, and modulators of enzymes of the NAD+ biosynthesis pathways [68]. As NAD+ is mainly produced by the NAD+ salvage pathway from niacinamide/nicotinamide, nicotinamide riboside (NR), and nicotinamide mononucleotide (NMN) [4,7,53,54,55,56], these seem to be the most promising NAD+ boosters, since more steps are required for nicotinic acid (NA) and tryptophan to form NAD+ [69]. NAD+ availability can be elevated by PARPs pharmacological inhibitors (BGB-290, olaparib, rucaparib, veliparib, CEP-9722, E7016, talazoparib, iniparib, niraparib (MK—4827), PJ34, DPQ, 3-aminobenzamide) [70,71] and CD38 inhibitors [72,73,74], such as flavonoids including epigenin, luteolin, quercetin, kuromanin, luteolinidin, or thiazoloquin(az)olinones such as the compound “78c” [72,75,76,77]. There are also chemical inhibitors of the NAD+ biosynthesis enzymes, such as FK866, an NAMPT inhibitor, gallotannin, an inhibitor of NMNAT and phthalic acid (PA), an inhibitor of QPRT [67,78] hydroxynicotinic acid, an inhibitor of NAPRT [79]. However, little is known regarding their pharmacology and safety for human use to increase NAD+. The use of PARP inhibitors might have detrimental effects as PARPs are involved in fundamental cellular process and PARP inhibition induces genomic instability [80]. The mentioned inhibitors, including CD38 homologue CD157, are currently investigated as drugs for cancer treatment [24], and the inhibition of CD38 and PARPs in non-oncogenic conditions is poorly explored so far. Activation of NAD+-generating enzymes by AMPK and NAMPT activators is another approach for boosting NAD+ levels [81,82]. Such activators are 5-aminoimidazole-4-carboxamide epigallocatechin gallate, leucine, metformin, resveratrol, ribonucleotides, P7C3, small molecule SBI-797812, and proanthocyanidins [81,83,84,85,86,87,88,89,90,91]. The most important NAD+-generating enzyme is NAMPT, which converts NAM to NMN [81]. NAMPT biosynthesis can be modulated by the small synthetic molecule activators P73C and SBI-797812 [81], or notoginseng leaf triterpenes and a natural peptide IRW (Ile-Arg-Trp), natural compounds that activate Nampt gene expression and/or increase intracellular NAMPT protein abundance [92,93,94]. Further studies are needed to evaluate the health risks and benefits of continuous use of NAMPT activators and PARP and CD38 inhibitors in humans for medical purposes to increase NAD+ bioavailability.

## 4. Safety Dose of NAD+ Precursors

WHO and the FAO recommend a daily intake of 11–12 mg of vitamin B3 (niacin), commonly known as nicotinic acid and nicotinamide, for adults [95]. NAD+ can be synthesized from the acid, amide, or riboside form of the vitamin B3 [96]. Based on numerous results from human clinical trials of nicotinic acid and nicotinamide, two bodies have established the highest daily dose for chronic ingestion that is unlikely to pose a risk of adverse health effects for almost everyone in the general population. The UK Expert Working Group on Vitamins and Minerals (EVM) [97] set a guideline for dietary supplementation with nicotinamide of 500 mg/day for a 60 kg adult, while the European Commission Scientific Committee on Food (EU SCF) set the tolerable upper intake level (UL) at 900 mg/day [98]. For preventing deficiency (pellagra), 16 mg of B3 for adult men and 14 mg for adult women are the recommended daily allowances (RDAs) [99]. Doses of nicotinic acid at 50 mg/day and higher are associated with flushing and itching that occur within 30 min after the oral administration [100]. Large doses of 250 mg per day of nicotinamide have induced reversible hepatotoxicity in animals and humans [101,102] or minor abnormalities of liver enzymes [103]. Although nicotinic acid and nicotinamide have been studied for a very long time, there are some issues regarding the older studies. For example, according to Knip and coworkers (2000), “Safety data have not been collected in a systematic manner and many older reports failed to distinguish between nicotinamide, nicotinic acid and combined vitamin regimens containing nicotinamide” [103]. NMN and NR on the other hand are relatively newly discovered forms of NAD+ precursors; consequently, there are fever studies on their toxicity and side effects. NR and NMN may be better tolerated compared to other nicotinamide adenine dinucleotide substrates since they do not trigger side effects, such as “flushing” or hepatotoxicity, which are characteristic for nicotinic acid, nor side effects of nicotinamide, including sirtuin inhibition [104]. No adverse effects (increased toxicity or increased mortality) were observed on wild-type mice after a 12-month administration of NMN, indicating its long-term safety [105]. A study assessing the safety of NR chloride at 300, 1000, and 3000 mg/kg/day over 14 and 90 days in rats revealed the lowest observed adverse effect level (LOAEL) for NR to be 1000 mg/kg/day, and the no observed adverse effect level (NOAEL) to be 300 mg/kg/day for NR [106]. The upper level (UL), which is by definition »the maximum level of the daily nutrient intake that is likely to result in no adverse effect« [99], was established for NR intake of 3 mg/kg/day (180 mg/day for a 60 kg adult). This was calculated from NOAEL by applying a 100-fold safety factor [106]. A few small human pharmacokinetics studies have been conducted on NR, primarily investigating pharmacokinetics in humans and the effects of treatment with NR on blood NAD+ levels. For example, NR was well-tolerated, as no severe side effects were reported by participants in two studies [107,108,109]. In a clinical trial with middle-aged human subjects, an oral dose of 1 g of NR once per day did not produce any drug-related adverse effects and clearly increased NAD+ levels (around 60% over the control group) in peripheral blood mononuclear cells [110].

There are more Phase 1 clinical trials with NR than with NMN. The ClinicalTrials.gov database lists (on date 25 July 2022) 73 trials for nicotinamide riboside, 10 trials for nicotinamide mononucleotide, 619 trials for niacin (including nicotinic acid, niacinamide, and nicotinamide), and 290 trials for tryptophan (Table 1).

The safety dose, therapeutic dose, therapeutic window, and optimal treatment duration for NAD+ precursors is still not known, especially in the case of NMN and NR. This information and the optimal therapeutic dose and treatment period of NAD+ precursors in various diseases should be determined in future human clinical trials.

## 5. Potential Safety Considerations/Concerns

Clinical trials on NR and NMN administration demonstrated their safety regarding toxicity and their ability to efficiently increase NAD+ levels in healthy volunteers so far [111]. NR was granted “Generally Recognised as Safe status” by the US Food and Drug Administration (FDA). NR has also been reviewed and approved by Health Canada, the European Food Safety Authority (EFSA), and the Therapeutic Goods Administration of Australia, although there may be some safety concerns, at least in mice. A high dose of dietary NR caused glucose intolerance and dysfunction of the white adipose tissue in mice fed a slightly obesogenic diet [112]. Melo and coworkers (2000), as well as Ramsey and coworkers (2008), reported impaired glucose metabolism in mice supplemented with NMN and NAM [113,114]. Currently there are no long-term human safety trials evaluating the safety of NAD+ boosters NR and NMN. Current data are based on small participant numbers of a few small-scale human clinical trials that implied that increased NAD+ levels from NAD+ boosters were safe in humans [69,107,108,110,115,116,117,118]. For example, in the blood of 12 subjects with a single oral dose of 1000 mg, NR increased NAD+ by 2.7-fold [107]. Oral ingestion of NR, 250–1000 mg/d for 9 days, resulted in a 2-fold increase in NAD+ in 140 healthy volunteers [108]. There are seven clinical trials on NR [107,108,110,117,119,120,121] and far fewer data on the safety and oral availability of NMN in humans. Only one clinical trial involving 10 volunteers receiving a single oral dose of 100–500 mg NMN indicated safety and efficacy of NMN, as no significant adverse effects were observed [116]. In a recent study, NMN supplementation increased muscle insulin sensitivity, insulin signaling, and remodeling in overweight or obese women with prediabetes [122]. Most side effects reported during NAM, NR, and NMN administration are minor (e.g., diarrhea, nausea, rashes, flushing, calf cramps, thrombocytopenia, erythema pruritis, skin burning, fatigue, abdominal discomfort, and headache) and are relatively infrequent [123,124]. Increased acetylcarnitine concentrations in skeletal muscle and minor changes in body composition and sleep pattern were reported in a recent study of NR supplementation in healthy obese individuals [125]. On the other hand, there have been more clinical trials evaluating the safety of NA and NAM, and the pharmacokinetics of NA and NAM have been studied in detail [96]. Supplemented NA can result in hot flashes and increased blood sugar as well as homocysteine levels in high doses. Overdose of nicotinamide (NAM) can cause hepatotoxicity in rare cases [103]. In NAM-treated rats, impaired glucose tolerance and increased hepatic and renal markers of oxidative DNA damage have been observed [126].

Evidence for health risk assessment is still limited, and long-term oral ingestion of NAD+ precursors NR and NMN has not yet been studied in long-term human clinical trials, or human clinical trials have not been completed. In addition, there are insufficient data on the elevation of NAD+ levels in various clinical conditions. Therefore, the long-term consequences of increasing NAD+ levels have yet to be investigated in-depth in human clinical trials.

## 6. Is Increased NAD+ Tumorigenic?

There are a few reports of genotoxicity of nicotinic acid, while NAM and NR were not found to be genotoxic [106]. Nicotinamide, NR, and NMN were also not found to be directly carcinogenic [23,127]. No evidence was found that treatment with NR or NMN for a prolonged period stimulated tumor development in animals [17,105] although further studies are warranted since ingestion of NAD+ boosters results in elevated NAD+ levels that could indirectly influence tumorigenesis. Indeed, levels of NAD+ are a crucial protective factor in early carcinogenesis and could later become a detrimental factor in the phases of cancer development and promotion. Specifically, maintenance of adequate NAD+ levels regulates redox homeostasis and mitochondrial metabolism, which are predisposed conditions for genome integrity and prevention of tumorigenesis [128]. However, in the phase of cancer progression and treatment, elevated NAD+ levels may have deleterious effects on the process of malignancy by promoting cell survival, encouraging growth, increasing resistance to radio- and chemotherapy, and promoting inflammation (reviewed in [24,129]) and stimulation of angiogenesis [130]. Although increased NAD+ levels can induce resistance to radiotherapy, nicotinamide (as a radiosensitizer) is administered as a strategy to improve the radio-sensitivity [131].

In contrast, it is feasible that restoration of NAD+ in the early stages of the carcinogenic process induces cellular repair and adaptive stress responses and regulates cell cycle arrest and apoptotic clearance of damaged cells, thus preventing or reversing the malignant phenotype (reviewed in [24]). For example, the potential pro-tumorigenic side effects of NAD+ precursor NMN were discussed by Nacarelli et al. (2019): NMN intake by mice increased NAD+ levels, which influenced the secretory activity of senescent cells to promote tumor progression of pancreatic and ovarian cancers [132]. In contrast, restoring NAD+ pools with NR prevented DNA damage and hepatocellular carcinoma formation in a mouse model of a liver cancer [27].

Increased NAMPT activity was observed in several human cancers [133], and NAMPT inhibition by drugs is promising as a cancer treatment. For example, FK866 has anticancer effect. It is an inhibitor of nicotinamide-recycling enzyme (NAMPT/PBEF) that catalyzes the rate-limiting step of NAD+ synthesis [134], resulting in apoptosis induction in a tumor due to NAD+ depletion [135]. Activity of sirtuins and PARPs is modulated by NAD+ levels. They have both procancer and anticancer effects [136,137,138,139,140,141]. Therefore, their role in cancer prevention and promotion must be fully elucidated in future studies. In conclusion, severe deficiency of NAD+ stops cancer growth, whereas an increase in NAD+ level might promote cellular NAD+ anabolism and accelerate tumor growth.

## 7. Undesirable Effects on Inflammation

Increased levels of NAD+ were reported to increase inflammation and aging [132]. Namely, increased NAD+ influences the inflammatory signaling of senescent cells in vivo in mouse models of pancreatic and ovarian cancers through the higher mobility group A (HMGA) proteins and NAMPT expression, which promotes the proinflammatory senescence-associated secretory phenotype (SASP) through NAD+-mediated suppression of AMPK kinase, leading to suppression of the p53-mediated inhibition of p38 MAPK and enhanced NF-κB activity [132]. In particular, CD38 or cyclicADP ribose hydrolase is on the surface of immune cells and is one of glycoproteins associated with pro-inflammatory SASP [142]. Its activity leads to NAD+ degradation and formation of cADP ribose from NAD+ and therefore possibly limiting the deleterious effect of NAD+ on SASP-mediated inflammation. NAD+ supplementation with the precursor NR for 5 months reduced and not increased the neuroinflammation in a transgenic mouse model of Alzheimer’s disease [143]. More senescent cells are formed with age increasing the SASPs. NAD+ worsens SASP-associated inflammation [142]. As the senescent cells also secrete CD38, NAD+ levels may decrease, perhaps forming a self-correcting system. Further studies are needed to assess the potential problem of increased inflammation caused by elevated NAD+ levels and to clarify the role of NAD+ in SASP regulation and mechanisms of regulation of CD38 expression in aging tissues.

## 8. The Potential Problem of Methylation

Methyl groups are important for the synthesis of creatine, choline, and other neurotransmitters, as well as for other biological functions. High dose supplementation with NAM, NR, and NMN may increase nicotinamide levels [144,145,146]. Excessive NAM is theoretically methylated to MeNAM, which could (i) lead to a deficiency of methyl donors (e.g., S-adenosyl methionine) for dopamine and creatine synthesis and (ii) increased risk of vascular disease neurodegenerative and chronic kidney disease by producing more homocysteine [147,148]. Interestingly, Conze et al. [117] observed no homcysteine increase in subjects consuming up to 1000 mg of Niagen (nicotinamide riboside chloride) over a 56-day period. Thus, further studies are needed to clarify the issue of methylation as a consequence of NR and NMN supplementation.

## 9. Potential (In)Direct Harmful Effects of Increased NAD+

It is difficult to detect and investigate indirect adverse health effects in animal studies and human clinical trials. Nevertheless, there are some reports of possible adverse effects. NA and NR decreased physical performance in young rats [149] and the capacity of high-intensity exercise in humans [150]. On the other hand, elderly people seem to benefit from dietary NR supplementation. Specifically, increased NAD(P)H levels, decreased oxidative stress, and improved physical performance were observed only in the elderly participants of the study [121]. Cardiopulmonary exercise performance was improved after 6 weeks of NMN supplementation due to enhanced O_2_ utilization of the skeletal muscle in 48 recreationally trained runners [151]. The Japanese authors claim in a pre-print publication that chronic oral supplementation of NMN for 12 weeks significantly improved muscle strength and performance in 10 volunteers [152]. Kourtzidis et al. [153] expressed concern that supplementation with compounds involved in redox homeostasis can lead to adverse effects in healthy young people, as their endogenous antioxidant protection is still functioning adequately. An increase in NAD+ levels could also have a negative effect on longevity. Overexpression of SIRT1 did not extend the lifespan of mice on a standard diet, although it improved overall health and decreased carcinomas [154]. Supplementation with NAM improved health span and did not alter lifespan in the mouse model [155].

As mentioned above, the increase in NAD+ levels by NAD+ precursors could also affect the process of cancerogenesis, because increased NAD+ levels have potentially both cancer-promoting and cancer-inhibiting effects. Indeed, NAD+ is a crucial protective factor in early cancer development and could become a detrimental factor later in the phase of cancer development and promotion [24,25]. None of the animal studies in which NMN or NR were supplemented reported increased cancer incidence. There is also controversy about the involvement of NAD+ in the activation of PARPs and sirtuins in carcinogenesis and inflammation/sepsis. Elevated NAD+ levels may have different roles in different stages of sepsis. Activation of SIRT1 has beneficial effect in the initial (proinflammatory) phase, characterized by a cytokine storm, overproduction of reactive oxygen species (ROS), and metabolic shift [156], while SIRT1 expression should be inhibited in the later stages of sepsis [157]. Therefore, the role of increased amounts of SIRT1 substrate, NAD+, cannot be simply defined as beneficial or detrimental in sepsis. Increase in NAD+ could also have negative effects in rheumatoid arthritis and other inflammatory diseases because of stimulation of inflammatory cytokine secretion by leukocytes [158]. Accumulation of toxic degradation products and metabolites of NAD+ precursors, like N-methyl-2-pyridone-5-carboxamide (2-PY), N-methyl nicotinamide (MeNAM), and nicotinic acid adenine dinucleotide (NAAD) is also potentially harmful [118,159]. Their increase were reported upon the NR supplementation [118,159]. NAM, NR, and NMN supplementation can increase NAM levels, which could inhibit PARPs and CD38 activities [160]. A potential inhibitory effect of NAM on sirtuins was hypothesized [161,162,163], although SIRT1 feedback inhibition by NAM may not play a significant role in vivo [164,165]. Possible adverse effects of a high-dose nicotinamide may alter the methyl pool that is used to methylate DNA and proteins [165] as discussed above. Additionally, it is also necessary to highlight the effect of nicotinamide N-methyltransferase (NNMT), the master regulator of intracellular NAM, on NAD+ levels and malignant transformation. NR and NMN may increase nicotinamide levels, thus enhancing the NNMT activity due to the higher substrate availability [166]. NNMT catalyzes the N-methylation of nicotinamide, using S-adenosyl-L-methionine (SAM) as a methyl donor, thus yielding N1-methylnicotinamide (MNA) as a product and releasing S-adenosyl-L-homocysteine (SAH) [167]. NNMT activity can thus affect NAD+ biosynthesis and ATP production, as well as drive epigenetic modifications and impact gene expression by modulating the intracellular SAM/SAH ratio [167]. Further, the enhanced NNMT activity and increased NNMT reaction products due to the conversion to nicotinamide from NAM, NR, or NMN supplementation might potentially be related to cancer development [166]. There is active research on discovering the inhibitors of NNMT enzyme activity with some small molecule inhibitors showing promising results [167,168,169,170,171,172,173]. Although NNMT may exert a primary role in the first step of carcinogenesis by irreversibly methylating NAM, thus generating MNA [166], the effective role of this enzyme as well as NAD+ boosters on cancer development still need to be fully elucidated.

## 10. NAD+ Supplementation and Circadian Rhythm

The NAD+ level naturally fluctuates in a 24 h rhythm and is altered by exercise, diet, sleep, and NAD+ boosters [174,175,176]. Thus, NAD+ supplements can affect the circadian clock, as the circadian rhythm of sleep and wakefulness is also controlled by NAD+ levels and sirtuin activity; NAD+ controls circadian reprogramming through the nuclear translocation of a PER2 protein [177]. Circadian rhythms are important for hormone release, eating habits, digestion, and body temperature in addition to sleep patterns. Therefore, it is important to maintain robust oscillations without deterioration of circadian rhythm (alteration of amplitude, period, and phase) by inappropriate timing of consumption of NAD+ precursors and other lifestyle choices [2,178]. How NAD+ extended-release boosters affect the 24 h clock oscillation and endogenous NAD+ levels is a question that remains to be answered.

## 11. At What Age Does It Make Sense to Start Taking NAD+ Boosters?

Although there are no human studies that address this question, it would be reasonable to wait to take NAD+ boosters until endogenous NAD+ levels begin to drop significantly at older ages and NAD+ metabolism becomes altered. As shown by the results of several studies, the improvements were greatest when the intake of NAD+ was started after the developmental period (e.g., midlife) in mice. For example, treatment of old mice with NAD+ precursors or PARP inhibitors improved health parameters including extension of lifespan, protection against metabolic disease, increased insulin sensitivity, reversal of mitochondrial dysfunction and reduced stem cell senescence [23,42,70,179]. The use of NAD+ boosters when cellular NAD+ levels are already/still adequate may thus be unwise [96]. Therefore, we need to establish the normal/healthy NAD+ levels and how to measure them. The age-related reference intervals for NAD+ levels should be determined in future clinical trials.

## 12. None, Continuous, or Intermittent NAD+ Supplementation?

Poljsak and colleagues [178] hypothesized that circadian rhythmicity was a mechanism that prevented disruption of the negative feedback loops of organismal homeostasis. Circadian rhythmicity inhibited the loss of the cell’s response to various endogenous substances, while the constant stimulus, like the constant concentration of endogenous substances, could have the opposite effect. Thus, problems could arise when people consume NAD+ boosters continuously or those with extended release and eliminate the intracellular circadian fluctuations of NAD+ during the 24 h period. It should be emphasized that NAD+ is required for over 500 enzymatic reactions in the human body [180], and it is estimated that the expression of one in ten genes is under the circadian control [181,182]. There are no scientific studies that prove or disprove to take NAD+ boosters at intervals. In the case of resveratrol (3,5,4′-trihydroxystilbene), a potent natural sirtuin activator, lifespan extension was observed in mice fed a high-fat diet only when the animals were treated every other day (intermittently) [62,183,184]. Such intermitted exposure seems necessary to prevent the resistance development. Dellinger et al. [120] observed that repeated administration of NR and pterostilbene increased NAD+ levels by 40% during continuous supplementation after 4 weeks in 60- to 80-year-old adults and observed no side-effect in 8 weeks of trial. 

Nevertheless, the concern remains that constant activation of sirtuins by NAD+ boosters is not beneficial. For example, mice with consistently elevated SIRT1 in the brain show increased anxiety; variations in the SIRT1 gene are also associated with an increased risk of anxiety in humans [185,186]. Since nicotinamide mononucleotide (NMN) is produced by the enzyme NAMPT, high concentrations of NMN may induce a “feedback inhibition effect” on NAMPT, inhibiting the formation of NAD+. Although this effect has not been proven so far, a similar NAM “feedback inhibition effect” has been observed in NAD-dependent processes such as PARP/Sirtuin/CD38 reactions [53,164]; in vivo PARP-1 activity inhibition by NAM remains arguable. As it has been reported that treatments with NMN and NR have beneficial health effects in various model organisms and in humans [17,22,23,33,40,105], the question arises whether the beneficial effects are even greater when NMN and NR are administered at intervals (e.g., every other day). Indeed, the continuous supply of NMN or NR could suppress the enzyme NAD(P)H dehydrogenase, quinone 1, which regulates the NAD/NADH ratio by oxidizing NADH to produce NAD+ [187]. In conclusion, the potential feedback suppression and adaptive response caused by elevated NAD+ levels and the role of elevated NAD+ concentrations on circadian rhythms should be carefully investigated in the future.

## 13. Role of Intestinal Microbiota in the Bioavailability of NAD+ Precursors

The gut microbiota produces hundreds of bioactive compounds, including B vitamins. Human digestion and the microbiome play a role in the provision of B3 vitamin forms, although their interaction has not yet been fully characterized [188]. Through the small intestine, both nicotinamide and nicotinic acid are directly absorbed where nicotinic acid is converted to nicotinamide [189]. Intestinal bacteria play an important role in NAD+ synthesis from tryptophan [188,190]. Different microorganisms that synthesize NAD have been identified [188]. Gut microbiota are involved in de novo biosynthesis pathway generating NAD+ from L-aspartate [191]. Certain gut microorganisms increase the biosyntheses of NAM in the presence of L-aspartate and L-tryptophan [192]. Gram-negative bacteria release circulating lipopolysaccharides (LPS), and the possible link between infection, inflammation, and CD38 was reported [193]. An important role of the gut microbiome in the assimilation of orally delivered NMN and evidence for indirect upregulation of the NAD+ metabolome was recently reported as a pre-print [194]. Studies on the relationship between gut flora and NAD+ boosters are sparse. Future studies should examine how specific NAD+ boosters affect the microbiota, how they regulate immune cell activity, how the gut microflora affects the uptake of NAD+ precursors, and how the gut environment affects the potential degradation of NAD+ precursors, as it has been suggested, for example, that NR may be degraded in the gut to NAM [195].

## 14. Which Precursor Is Better?

All NAD+ precursors: NMN, NR, nicotinic acid, and tryptophan increase the availability of NAD+ [6,7,8,9]. As noted above, the precursors differ in their ability to enhance NAD+ synthesis via their position in the NAD+ assembly pathway and differ in the number of steps required to form NAD+ in the biochemical pathways. There is a need for more data on precursor differences in absorption, bioavailability, distribution, metabolism, excretion, tissue specificity, and efficacy in increasing NAD+ levels in humans. Currently, there are no studies that directly compare the efficiency of the different precursors to conclude which of the NAD+ boosters has the best therapeutic prospects. Intravenous administration of NR and NMN to compare their efficiency to convert to NAD+ was only investigated in peripheral rodent tissues [59].

## 15. Which Age-Associated Diseases Could Be Treated with NAD+ Boosters?

The therapeutic potential of dietary NAD+ boosters could improve the impaired NAD+ availability that may promote the development of age-related, cardiovascular, sarcopenic, menopausal, metabolic, inflammatory, renal, neurodegenerative, muscle-damaging, and mitochondrial diseases, cancers, and COVID-19 [68]. Animal studies revealed that NAD+ booster supplementation enhanced life span [17] and improved cardiovascular function [33] enhanced muscle regeneration [17], improved mitochondrial and stem-cell function [179], and altered glucose metabolism [23]. Detailed human studies should investigate which age-related degenerative diseases can be treated, delayed, or prevented with NAD+ boosters, and which booster, which treatment duration, and which dosing regimen is the best. Since most human studies have been conducted on “healthy” individuals, patients with certain diseases should also be included in clinical studies in the future.

## 16. Conclusions and Perspectives

Results from preclinical studies and preliminary results from small human clinical trials imply that promoting intracellular NAD+ anabolism through NAD+ boosters is a promising therapeutic strategy for age-associated degenerative diseases [96]. Little is known about the pharmacokinetics, efficacy, safety, and potential side effects of long-term supplementation with NAD+ precursors. There are many unanswered questions, due to the limited number of human trials and lack of clinical data. Future clinical trials should focus on the safety and risk assessment of NAD+-boosting therapies, as there is some evidence of direct and indirect adverse effects. Human studies with larger participant numbers are therefore desired to evaluate long-term toxicological outcomes. It is difficult to extrapolate or transfer results from animal models to humans because of variations in metabolism, longevity, and genetic variability. Current knowledge of the beneficial effects of NAD+ on aging and healthspan is primarily based on cell-culture experiments and model organisms. There are few human studies that have examined key outcomes such as increased vitality, reduction in all causes of death, prolonged health, and lifespan. Further in-depth studies and clinical trials are needed to understand the fundamental aspects of NAD+ biology and physiology and to clarify the use of NAD+ precursors for prevention or treatment and to promote health and longevity. 

The effects of various NAD+ boosters in different disorders should be additionally investigated. Special attention should be given to study synergistic/antagonistic effects between NAD+ precursors, CD38 and/or PARP1 inhibitors, and the use of other drugs and supplements. Data from human studies may be biased by lifestyle factors such as diet, exercise, age, and genetic factors. Special attention should also be paid to interindividual variations/differences in NAD+ amounts of participants at baseline in clinical trials. Need for NAD+ supplementation should be precisely determined. Potential serious risks such as tumor development and progression, adverse effects on inflammation, senescence, circadian rhythm disruption, and feedback inhibition should be additionally investigated, as well as the potential hormetic effect of increased NAD+ where a low-to-moderate dose might stimulate beneficial effects, and a high (over)dose might induce an inhibitory or even toxic effect. For example, supraphysiological dose of NAM was shown to decrease lifespan in *C. elegans* and yeast [19,144,145]. In this regard, special attention is needed in supplementation with NAD+ precursors NR and NMN, which induce high levels of NAM to prevent undesirable effects [146]. Since the effects of NAD+ occur via the regulation of DNA repair, stress responses, and energy metabolism, it seems worthwhile to test NAD+ precursors in a manner of drug development to better understand the therapeutic role of NAD+ precursors in the prevention and treatment of human disease.

## Figures and Tables

**Table 1:** Information on clinical trials regarding NAD+ precursors: NR, NMN, niacin (nicotinic acid and niacinamide/nicotinamide) and tryptophan.

NAD+ Precursor	Number of Studies						
	Not Recruiting	Recruiting	Active	Terminated	Completed	Suspended	Unknown Status
**NR**	3	10	4	1	24	2	8
**NMN**	0	5	2	0	7	0	0
**Niacin**	22	110	24	39	357	5	47
**Tryptophan**	16	57	14	21	131	0	38
